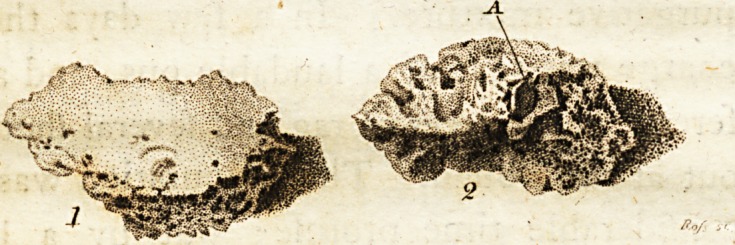# An Account of a Case in Which the Head of the Os Femoris, Shattered by a Gun Shot, Is Supposed to Have Been Regenerated

**Published:** 1786

**Authors:** Joseph Brandish

**Affiliations:** Surgeon, at Alcester, in Warwickshire


V. *An Account of a Case in which the Head of the Os Femoris, shattered by a Gun Shot, is supposed to have been regenerated. By Mr.* Joseph Brandish, *Surgeon, at Alcester, in Warwickshire. Communicated, with an Account of a curious Fact relative to the Effects of Opium, in a Letter to Dr.* Simmons, *by* James Johnstone, *M. D. Physician at Worcester.*

To Dr. Simmons.

SIR,

I TAKE the liberty to communicate to you the inclosed case, by Mr. Brandish, a very deserving surgeon and apothecary at Alcester, to be inserted, if you think proper, in the London Medical Journal. I have lately had an opportunity of examining the patient, and had the pleasure to find that the wounded limb seems hardly shorter than the other, and that he can bend and extend it, so as to be able to walk with tolerable ease.

This case, considering the quantity of bone that came away, may serve as an extraordinary and happy instance of the powers of nature in reproducing a part of one of the largest bones,

 and and in healing one of the moft defperate wounds, when affifted by a diligent and fkilful furgeon.

On the fubject of this cafe I have only farther to add, that the abdomen having received no immediate injury from the gun fhot, was affected only in confequence of its vicinity to the wound, the inflammation and irritation of the hip having occafioned pain, for fome time, both in the bladder and inteftines.

Mr. Brandifh has lately mentioned to me another fact, which I embrace this opportunity of communicating to you, as being an addition to the inftances which fhew that the torture of pain refifts the fedative power of opium; and that the reft and eafe procured by the latter, when adminiftered even in the largeft dofes, are only gradually effected, efpecially when the pain is exceffive. The fact I allude to is as follows: — In the year 1776, a boy at Alcefter, aged fourteen years, took from half to three quarters of an ounce of the Thebaic tincture every other night for three weeks, previous to his undergoing the operation of lithotomy in the Worcefter Infirmary. The whole quantity of tincture taken in this fpace of time amounted to thirteen ounces. The firft night after each dofe he had no fleep; but he ufually flept the following following night. Mr. Bagnall took from his bladder a rough ſtone of the ſize of a large mulberry, and he recovered, and has remained well ever ſince.

I am now preparing conſiderable additions and illuſtrations for a new edition of my tract on the uſes of the ganglions of the nerves. Almoſt all the late anatomical works, particularly thoſe of Wriſberg, Walter, and Monro, afford new proofs of the certainty of the doctrine I have laid down. This certainty, and ſome very important applications of the doctrine in queſtion, I ſhall endeavour to demonſtrate, as I firmly believe it to be one of the moſt important pieces of knowledge we have concerning the nervous ſyſtem; next, indeed, in importance, and equal in certainty to this, that the nerves are the media of ſenſation and motion.

I am,

SIR,

Your faithful and

Moſt obedient ſervant,

*Worceſter,*
April 26, 1786.

J. JOHNSTONE.

CASE.

### CASE.

On the 23d of December, 1783, I was defired to vifit James Whiffill, a lad about twelve years of age. The account I received of him was, that having carelefly put the breech of a gun in the fire, in order to clean it, and the gun happening to be loaded, the charge went off as foon as the gun was fufficiently heated, and the whole contents of the piece paffed into the upper part of one of his thighs, adjoining to the middle of the groin, and came out about the middle of the glutæus maximus.

The orifice in the groin might have been covered with a fhilling, that part of the patient's body having been clofe to the muzzle of the gun; but the orifice in the buttock was larger than half a crown.

As I had fucceeded not long before by means of a feton, in the cafe of a young man who was fhot through the thick part of his thigh, I was defirous of adopting the fame mode of treatment in the prefent cafe; but I here found the introduction of a feton impracticable; I therefore contented myfelf with injecting fpirit of turpentine and linſeed oil, warm, into the wound by means of a ſyringe, and afterwards covering the wounds with a large poultice of bread and milk. At the ſame time I directed the patient to take frequently of a purging mixture till ſtools were procured; and in the evening, as he complained of great pain in his bowels, and about the region of the bladder, I ordered him to be well fomented with a decoction of green chamomile flowers. The next morning he had ſtools, and made water.

December 26th, I repeated the uſe of the purgative mixture. In a few days the diſcharge was that of a laudable pus, and at different times pieces of rag and ſeveral ſhot came out at the wound. The ſuppuration was for a conſiderable time profuſe; but by a liberal uſe of the bark, port wine, &c. the patient's ſtrength was ſupported.

I dreſſed him every day till the 8th of May, 1784, and after that occaſionally till the month of September following. During the courſe of this attendance, ſeveral ſucceſſive abſceſſes were formed, and ſeveral exfoliations of bone came away; one in particular, which appeared to be a conſiderable portion of the head of the thigh thigh bone, with a ſhot ſticking in it *. The wounds have been for ſome time perfectly heal-ed, and the boy is now in good health, and walks tolerably well with the aſſiſtance of a crutch.

*Alceſter,*
October 5, 1785.

* Of this portion of the head of the bone Dr. Johnſtone having cauſed an engraving to be made, has obligingly favoured us with the uſe of the plate. — Fig. 1 repreſents the outer ſurface of the bone, and fig. 2, the ſame piece of bone inverted, to ſhew (A) a lead ſhot ſticking in it.—EDITOR.

VI. *An*

## Figures and Tables

**Figure f1:**